# Refinements and generalizations of some inequalities of Shafer-Fink’s type for the inverse sine function

**DOI:** 10.1186/s13660-017-1554-1

**Published:** 2017-11-03

**Authors:** Branko Malešević, Marija Rašajski, Tatjana Lutovac

**Affiliations:** Faculty of Electrical Engineering, University of Belgrade, Bulevar kralja Aleksandra 73, Belgrade, 11000 Serbia

**Keywords:** 33B10, 26D05, sharpening, generalization, Shafer-Fink’s double inequality, arc sine function

## Abstract

In this paper, we give some sharper refinements and generalizations of inequalities related to Shafer-Fink’s inequality for the inverse sine function stated in Theorems 1, 2, and 3 of Bercu (Math. Probl. Eng. 2017: Article ID 9237932, 2017).

## Introduction

Inverse trigonometric functions, particularly the inverse sine function, have many applications in computer science and engineering. They are widely used in many fields, such as telecommunications, especially optical fiber telecommunications, signal processing, machine learning, and so on.

The main objective of the research presented in this paper is a refinement of Shafer-Fink’s inequality

1 $$ \frac{3x}{2+\sqrt{1-x^{2}}} \leq\operatorname{arcsin} x \leq\frac {\pi x}{2+\sqrt{1-x^{2}}} $$

for $x \in [0, 1]$; see [2, 3].

Various improvements of Shafer-Fink’s inequality have been considered so far in [4] and [5–12]. Also, let us mention that one refinement of Shafer-Fink’s inequality was given in [13], and it had applications in [14, 15] (see also [16]).

In this paper, we focus on the results of Bercu [1] related to Shafer-Fink’s inequality and give generalizations and refinements of the inequalities stated in Theorems 1, 2, and 3 in that paper. For e convenience of the reader, we further cite them.

### Statement 1

([1, Theorem 1])

*For every real number* $0 \leq x\leq1$, *the following two*-*sided inequality holds*:

2 $$ \frac{x^{5}}{180} + \frac{x^{7}}{189} \leq\operatorname{arcsin}x - \frac{3x}{2 + \sqrt{1 - x^{2}}} \leq\frac{\pi- 3}{2}. $$

### Statement 2

([1, Theorem 3])

*For every* $x \in [0,1]$ *on the left*-*hand side and every* $x \in [0, 0.871433]$ *on the right*-*hand side*, *the following inequalities hold*:

3 $$ \biggl( 1 - \frac{\pi}{3} \biggr) x + \biggl( \frac{1}{6} - \frac{\pi}{18} \biggr)x^{3} \leq\operatorname{arcsin} x - \frac{\pi x}{2 + \sqrt{1 - x^{2}}} \leq \biggl( 1 - \frac{\pi}{3} \biggr)x. $$

### Statement 3

([1, Theorem 2])

*For every* $0 \leq x \leq1$, *we have*:

4 $$ \operatorname{arcsin}x - \frac{3x}{2 + \sqrt{1 - x^{2}}} \geq \frac{a(x)}{2 + \sqrt{1 - x^{2}}}, $$

*where* $a(x) = (1/60)x^{5} + (11/840)x^{7}$.

## Main results

The main results of this paper are generalizations and improvements of the inequalities related to Shafer-Fink’s inequalities given in Theorems 1, 2, and 3 by Bercu [1], here Statements 1, 2, and 3.

First, let us recall some well-known power series expansions.

For $\vert x \vert \leq1$,

5 $$ \operatorname{arcsin}x = \sum_{m=0}^{\infty}{A(m) x^{2m+1}}, $$

where

6 $$ A(m)=\frac{(2m)!}{(m!)^{2} (2m+1)2^{2m}} $$

for $m \in N_{0}$.

Also, for $\vert x \vert \leq1$,

7 $$ \sqrt{1-x^{2}} = \sum_{m=0}^{\infty}{B(m) x^{2m+2}}, $$

where

8 $$ B(m) = \sum_{k=0}^{m}{\frac{2k!}{k!(k+1)!2^{2k+1}}} $$

for $m \in N_{0}$.

### Refinements of the inequalities in Statements 1 and 2

Let us consider the function

9 $$ \varphi_{k}(x) = \frac{k x}{2 + \sqrt{1 - x^{2}}} $$

for $x \in [0,1]$ and $k = 3$ or $k = \pi$.

Then, for $x \in [0,1]$, we have:

10 $$ \begin{aligned}[b] \varphi_{k}(x) & = kx \bigl(2 - \sqrt{1-x^{2}} \bigr) \cdot\frac{1}{3+x^{2}} \\ & = kx \Biggl(2 - \sum_{i=0}^{\infty}{B(i) x^{2i+2}} \Biggr) \cdot \sum_{j=0}^{\infty}{\frac{(-1)^{j}}{3^{ j+1}} x^{2j}} \\ & = \sum_{m=0}^{\infty}{C_{k} (m) x^{2m+1}}, \end{aligned} $$

where

11 $$ C_{k}(m) = \frac{(-1)^{m}k}{3^{m+1}} + \sum _{i = 0}^{m - 1}{ \frac{k{{(-1)}^{m-1-i}}(2i)!}{3^{m-i}i!(i+1)!2^{2i + 1}}} $$

for $m \in N$ and $C_{k} (0)=\frac{k}{3}$. Equality (11) is obtained by applying Cauchy’s product to the corresponding series.

It is easy to verify that the following recurrence relations hold:

12 $$\begin{aligned}& A(m+1) = \frac{(2m + 1)^{2}}{(2m + 2)(2m + 3)}A(m), \end{aligned}$$

13 $$\begin{aligned}& C_{k} (m+1) =\frac{k}{3} \frac{(2m)!}{m!(m+1)!2^{2m+1}} - \frac {1}{3}C_{k} (m), \quad C_{k} (0)= \frac{k}{3}, \end{aligned}$$

and

14 $$ C_{k}(m+1) = \frac{k}{3} \cdot \frac{2m + 1}{2m + 2}A(m) - \frac{1}{3}{C_{k}}(m) $$

for $m \in N_{0}$ and $k = 3$ or $k = \pi$.

Next, let us consider the function

15 $$ f_{k}(x) = \operatorname{arcsin}x - \varphi_{k}(x) $$

for $x \in [0,1]$ and $k = 3$ or $k = \pi$. Then, for $x \in [0,1]$, we have:

16 $$ f_{k}(x) = \sum_{m=0}^{\infty}{D_{k}(m) x^{2m+1}}, $$

where

17 $$ D_{k}(m) = A(m) - C_{k}(m) $$

for $m \in N_{0}$.

Let us prove that $D_{k} ( n ) > 0$ for all $n \in N$, $n \ge2$.

First, we note that for $k = 3$ or $k = \pi$, we have:

$$\begin{aligned} &D_{k}(2) = A(2) - C_{k}(2) = \frac{3}{40} - \frac{5k}{216} > 0, \\ &D_{k}(3) = A(3) - C_{k}(3) = \frac{5}{112} - \frac{17k}{1\text{,}296} > 0. \end{aligned} $$

Now, let us assume that the statement holds for $n=m$, that is, $D_{k} ( m ) > 0$.

We will prove that the statement holds for $n=m+2$, that is, $D_{k} ( m+2 ) > 0$.

Using the recurrence relations (12), (13), and (14), we get:

$$\begin{aligned} &D_{k}(m+2) \\ &\quad = A(m+2) - C_{k}(m+2) \\ &\quad = \frac{(2m+3)^{2}}{(2m+4)(2m+5)}A(m+1) - \frac{k}{3} \frac{{2m+3}}{{2m+4}}A(m+1) + \frac{1}{3} C_{k}(m+1) \\ &\quad = \biggl( \frac{(2m + 3)^{2}}{(2m + 4)(2m + 5)} - \frac{k}{3} \frac{2m+3}{2m+4} \biggr) A(m+1) + \frac{1}{3} \biggl( \frac{k}{3} \frac{{2m+1}}{{2m+2}}A(m) - \frac{1}{3}{C_{k}}(m) \biggr) \\ &\quad = \biggl( \biggl( \frac{{{{(2m+3)}^{2}}}}{{(2m+4)(2m+5)}} - \frac{k}{3} \frac{{2m+3}}{{2m+4}} \biggr) \frac{{{{(2m+1)}^{2}}}}{{(2m+2)(2m+3)}} + \frac{k}{9} \frac{{2m+1}}{{2m+2}} - \frac{1}{9} \biggr) A(m)\\&\qquad{} + \frac{1}{9}D_{k} ( m ) \\ &\quad = \frac{-13+5k+(50 - 8k)m+(136 - 44k)m^{2}+(64 - 16k)m^{3}}{36(m+1)(m+2)(2m+5)}A(m) + \frac{1}{9}D_{k} ( m ) \\ &\quad = \frac{k m^{2} (m - 1) + 5k - 13 + (50 - 8k)m + (136 - 43k)m^{2} + (64 - 17k)m^{3} }{36(m+1)(m+2)(2m+5)} A(m)\\&\qquad{} + \frac{1}{9}D_{k} ( m ). \end{aligned} $$

Observing the above expression and using the induction hypothesis ($D_{k} ( m )>0 $), we conclude that $D_{k} ( m+2 )>0$. Hence, by the principle of mathematical induction it follows that $D_{k} ( n ) > 0$ for all $n \in N$, $n \ge2$, that is,

18 $$ D_{k}(m) = \frac{(2m)!}{(m!)^{2} (2m+1)2^{2m}} - \Biggl( \frac{(-1)^{m}k}{3^{m+1}} + \sum _{i = 0}^{m - 1}{ \frac{k{{(-1)}^{m-1-i}}(2i)!}{3^{m-i}i!(i+1)!2^{2i + 1}}} \Biggr) > 0. $$

Thus, we have proved the following theorem.

#### Theorem 1

*For* $x \in [0,1]$, $n \in N$, *and* $k = 3$ *or* $k = \pi$, *we have the inequality*

19 $$ \sum_{m=0}^{n}{D_{k}(m) x^{2m+1}} \leq \operatorname{arcsin}x - \frac{kx}{2+\sqrt{1-x^{2}}}. $$

#### Remark 1

For $n = 3$ and $n = 1$, we get the left-hand sides of the inequalities stated in Statements 1 and 2, respectively (Theorems 1 and 3 from Bercu [1]).

#### Example 1

For $k=3$, the following statements are true for every $x \in [0,1 ]$.

- If $n=4$, then
  $$\frac{1}{180}x^{5} + \frac{1}{189}x^{7} + \frac{23}{5\text{,}184}x^{9} \leq \operatorname{arcsin}x - \frac{3x}{2 + \sqrt{1 - x^{2}}} \leq \frac{\pi- 3}{2}. $$
- If $n=5$, then
  $$\frac{1}{180}x^{5} + \frac{1}{189}x^{7} + \frac{23}{5\text{,}184}x^{9} + \frac{629}{171\text{,}072}x^{11} \leq \operatorname{arcsin}x - \frac{3x}{2 + \sqrt{1 - x^{2}}} \leq \frac{\pi-3}{2}. $$
- If $n=6$, then
  $$\begin{aligned} \frac{1}{180}x^{5} + \frac{1}{189}x^{7} + \frac{23}{5\text{,}184}x^{9} + \frac{629}{171\text{,}072}x^{11} + \frac{14\text{,}929}{4\text{,}852\text{,}224}x^{13} &\leq \operatorname{arcsin}x - \frac{3x}{2 + \sqrt{1 - x^{2}}} \\ &\leq \frac{\pi-3}{2}, \end{aligned} $$
  etc.

Also, for $k=\pi$, the following statements are true for every $x \in [0,1 ]$.

- If $n=2$, then
  $$\biggl(1 - \frac{\pi}{3} \biggr) x + \biggl(\frac{1}{6} - \frac{\pi}{18} \biggr) x^{3} + \biggl(\frac{3}{40} - \frac{5\pi}{216} \biggr)x^{5} \leq \operatorname{arcsin} x - \frac{\pi x}{2 + \sqrt{1 - x^{2}}} \leq \biggl(1 - \frac{\pi}{3} \biggr)x. $$
- If $n=3$, then
  $$\begin{aligned} &\biggl(1 - \frac{\pi}{3} \biggr)x + \biggl(\frac{1}{6} - \frac{\pi}{18} \biggr)x^{3} + \biggl(\frac{3}{40} - \frac{5\pi}{216} \biggr)x^{5} + \biggl(\frac{5}{112} - \frac{17\pi}{1\text{,}296} \biggr)x^{7} \\ &\quad \leq \operatorname{arcsin} x - \frac{\pi x}{2 + \sqrt{1 - x^{2}}} \leq \biggl(1 - \frac{\pi}{3} \biggr)x. \end{aligned} $$
- If $n=4$, then
  $$\begin{aligned} &\biggl(1 - \frac{\pi}{3} \biggr)x + \biggl(\frac{1}{6} - \frac{\pi}{18} \biggr)x^{3} + \biggl(\frac{3}{40} - \frac{5\pi}{216} \biggr)x^{5} + \biggl(\frac{5}{112} - \frac{17\pi}{1\text{,}296} \biggr)x^{7} \\ &\quad + \biggl(\frac{35}{1\text{,}152} - \frac{269\pi}{31\text{,}104} \biggr)x^{9}\leq \operatorname{arcsin} x - \frac{\pi x}{2 + \sqrt{1 - x^{2}}} \leq \biggl(1 - \frac{\pi}{3} \biggr)x, \end{aligned} $$
  etc.

### Refinements of the inequality in Statement 3

In [1, Theorem 2], Bercu proved the following inequalities for every $x \in [0, 1]$:

20 $$ \operatorname{arcsin}x - \frac{3}{2 + \sqrt{1 - x^{2}}} \geq \frac{a(x)}{2 + \sqrt{1 - x^{2}}}, $$

where $a(x) = (1/60)x^{5} + (11/840)x^{7}$.

We propose the following improvement and generalization of (20).

#### Theorem 2

*If* $n \in N$ *and* $n \geq2$, *then*

21 $$ \operatorname{arcsin}x - \frac{3x}{2 +\sqrt{1-x^{2}}} \geq \frac{\sum_{m=2}^{n}{E(m)x^{2m+1}}}{2 + \sqrt{1-x^{2}}} $$

*for every* $x \in [0,1]$, *where*

22 $$ E(m) = \frac{m (2m - 1)!}{(2m+1)2^{2m-2} m!^{2}} - \frac{2m 2^{2m-2}(m - 1)!^{2}}{(2m+1)!},\quad m \in N, m \geq2. $$

#### Remark 2

Note that inequality (20) is a particular case of (21) for $n = 3$.

#### Example 2

For $n > 3$, inequality (21) refines inequality (20), and we have the following new results.

- Taking $n=4$ in (21) gives
  $$\operatorname{arcsin}x - \frac{3x}{2 + \sqrt{1-x^{2}}} \geq \frac{ \frac{1}{60}x^{5} + \frac{11}{840}x^{7} + \frac{67}{6\text{,}720} x^{9}}{2 + \sqrt{1-x^{2}}} \quad \mbox{for all } x \in [0,1]. $$
- Taking $n=5$ in (21) gives
  $$\operatorname{arcsin}x - \frac{3x}{2 + \sqrt{1-x^{2}}} \geq \frac{ \frac{1}{60}x^{5} + \frac{11}{840}x^{7} + \frac{67}{6\text{,}720}x^{9} + \frac{3\text{,}461}{443\text{,}520} x^{11}}{2 + \sqrt{1-x^{2}}}\quad \mbox{for all } x \in [0,1]. $$
- Taking $n=6$ in (21) gives
  $$\begin{aligned} &\operatorname{arcsin}x - \frac{3x}{2 + \sqrt{1-x^{2}}}\\ &\quad \geq \frac{ \frac{1}{60}x^{5} + \frac{11}{840}x^{7} + \frac{67}{6\text{,}720} x^{9} + \frac{3\text{,}461}{443\text{,}520} x^{11} + \frac{29\text{,}011}{4\text{,}612\text{,}608} x^{13}}{2 + \sqrt{1-x^{2}}} \quad \mbox{for all } x \in [0,1], \end{aligned} $$
  etc.

#### Proof of Theorem 2

Based on Cauchy’s product of power series (5) and (7), the real analytical function

23 $$ g(x) = \bigl(2 + \sqrt{1-x^{2}} \bigr) \cdot \operatorname{arcsin}x - 3x $$

has the power series

24 $$ g(x) = \sum_{m=2}^{\infty}{E(m) x^{2m+1}} \quad \mbox{for } x \in[0,1], $$

where

25 $$ \begin{aligned}[b] E(m) &= \frac{3(2m)!}{m!^{2}(2m+1)2^{2m}} \\&\quad{}- \sum_{k=0}^{m-1}{\frac{(2k)!}{k! (k+1)! 2^{2k+1}} \cdot \frac{(2(m - k - 1))!}{((m - k - 1)!)^{2} (2m - 2k - 1) 2^{2(m-k-1)}}} \end{aligned} $$

for $m = 2, 3, \ldots$ .

First, we prove relation (22). Consider the sequence $(\mathsf{S}(m))_{m \in N, m\geq2}$ where

26 $$ \mathsf{S}(m) = \sum_{k=0}^{m-1} \mathsf{F}(m,k) $$

and

$$\mathsf{F}(m,k) = \frac{(2k)! (2(m - k - 1))!}{k! (k+1) !2^{2k+1} ((m - k - 1)!)^{2} (2m - 2k - 1) 2^{2(m-k-1)}}. $$

Consider the function^1^

$$\mathsf{G}(m,k) = \frac{(m-k) (4k^{2}-(6m+2)k-m) (2k)! (2m-2k-1)! }{(2m-2k+1) ((m-k)!)^{2} 4^{m} (k!)^{2}} $$

for $m \in N$ and $k \in \{0, 1, \ldots, m-1\}$. It is not hard to verify that the functions $\mathsf{F}(m,k)$ and $\mathsf{G}(m,k)$ satisfy the following relation:

27 $$ -2(m+1) m^{2} \mathsf{F}(m,k)+m(2m+3) (m+1) \mathsf{F}(m+1,k) = \mathsf{G}(m,k+1) - \mathsf{G}(m,k). $$

If we sum both sides of (27) from $k = 0$ to $k = m - 2$, then we get the relation

$$\begin{aligned} &-2(m+1) m^{2} \mathsf{S}(m)+m(2m+3) (m+1) \mathsf{S}(m+1) \\ &\quad = \mathsf{G}(m,m-1) - \mathsf{G}(m,0) - 2(m+1)m^{2}\mathsf {F}(m,m-1) \\ &\qquad {}+m(2m+3) (m+1) \mathsf{F}(m+1, m-1) + m(2m+3) (m+1) \mathsf{F}(m+1, m). \end{aligned} $$

Finally, as

$$\begin{gathered}\mathsf{G}(m,m-1)= -\frac{1}{3} \frac{(2m^{2}+5m-6) (2m-2)!}{4^{m} ((m-1)!)^{2}}, \qquad \mathsf{G}(m,0)=-\frac{m^{2}(2m-1)!}{(2m+1) 4^{m} m!^{2}}, \\ \mathsf{F}(m+1, m-1) = \frac{1}{6} \frac{(2m-2)!}{(m-1)! m! 2^{2m-1}}, \quad \quad \mathsf{F}(m+1, m) = \frac{(2m)!}{m! (m+1)! 2^{2m+1}}, \end{gathered} $$

and

$$\mathsf{F}(m, m-1) = \frac{(2m-2)!}{(m-1)! m! 2^{2m-1}}, $$

we have the following recurrence for $\mathsf{S}(m)$:

28 $$ -2(m+1) m^{2} \mathsf{S}(m) + m (2m+3) (m+1) \mathsf{S}(m+1) = \frac{(2m-1)!}{(2m+1) 4^{m} ((m-1))!^{2}}. $$

An algorithm for finding solutions of linear recurrence equations with polynomial coefficients can be found, for example, in [17] and [18].

It is easy to verify that the function

29 $$ \mathsf{S}(m) = \frac{1}{2} \frac{4^{m} m!^{2}}{(2m)!(2m+1)m}+ \frac {(2m)!}{4^{m} m!^{2} (2m+1) }, \quad m \in N, $$

satisfies the recurrence relation (28). Hence, based on (25), (26), and (29), we have:

30 $$ \begin{aligned}[b] E(m) & = \frac{3(2m)!}{m!^{2}(2m+1)2^{2m}} - \mathsf{S}(m) \\ & = \frac{m (2m - 1)!}{(2m+1) 2^{2m-2} m!^{2}} - \frac{2m 2^{2m-2}(m - 1)!^{2}}{(2m+1)!} \\ & = 2 \frac{(2m-1)!^{2} - m 2^{4m-4} (m-1)!^{4}}{2^{2m-2} (m-1)!^{2} (2m+1)!} \end{aligned} $$

for $m \in N$, $m \geq 2$.

Now we prove that $E(m) > 0$ for every $m=2,3, \ldots$ . It suffices to show that

31 $$ (2m-1)!^{2} - m 2^{4m-4} (m-1)!^{4} > 0 \quad\text{for } m \in N, m \geq2, $$

that is,

32 $$ T(m) = \frac{(2m-1)!^{2}}{ m 2^{4m-4} (m-1)!^{4}} > 1 \quad\text{for } m \in N, m \geq2. $$

Statement (32) is true for $m=2$, that is, $T(2) = 4 > 1$. Observing that

33 $$ T(k+1) = T(k) \frac{(2k+1)^{2}}{4 k (k+1)} $$

and using the induction hypothesis (i.e., $T(k) = \frac{(2k-1)!^{2}}{k 2^{4k-4} (k-1)!^{4}} > 1$ for some positive integer $k \geq 2$), we conclude, by the principle of mathematical induction, that $T(k+1) > 1$. Therefore, inequalities (32) and (31) are true, and consequently $E(m)>0$ for $m \in N$, $m \geq 2$. □

## Conclusion

In this paper, we proposed and proved new inequalities, which present refinements and generalizations of inequalities stated in [1], related to Shafer-Fink’s inequality for the inverse sine function.

Also, our approach provides inequalities that allow new approximations of the functions

$$\operatorname{arcsin} x - \frac{3x}{2+\sqrt{1-x^{2}}} \quad\mbox{and}\quad \operatorname{arcsin} x - \frac{\pi x}{2+\sqrt{1-x^{2}}} \quad\mbox{for all } x \in[0,1]. $$

Finally, let us note that proofs of inequalities (19) and (21) for any fixed $n\in N$ and $k \in\{3, \pi\}$ can be obtained by substituting $x = \sin{t}$ for $t \in[0, \pi/2]$ and using the methods and algorithms developed in [19] and [20].

## Footnotes

1 A method for determining function $\mathsf{G}(m,k)$ for a given function $\mathsf{F}(m,k)$ can be found in [17]. Note that the pair of discrete functions $(\mathsf{F}(m,k), \mathsf{G}(m,k) )$ is the so-called Wilf-Zeilberger pair [21].
